# The Effect of Vaginal Sildenafil on The Outcome of Assisted Reproductive Technology Cycles in Patients with Repeated Implantation Failures: A Randomized Placebo-Controlled Trial

**DOI:** 10.22074/ijfs.2020.5681

**Published:** 2019-11-11

**Authors:** Ashraf Moini, Fatemeh Zafarani, Nadia Jahangiri, Shahideh Jahanian Sadatmahalleh, Marya Sadeghi, Mohammad Chehrazi, Firoozeh Ahmadi

**Affiliations:** 1 Department of Endocrinology and Female Infertility, Reproductive Biomedicine Research Center, Royan Institute for Reproductive Biomedicine, ACECR, Tehran, Iran; 2 Department of Obstetrics and Gynaecology, Tehran University of Medical Sciences, Tehran, Iran; 3 Department of Obstetrics and Gynaecology, Arash Women's Hospital, Tehran University of Medical Sciences, Tehran, Iran; 4 Department of Reproductive Imaging, Reproductive Biomedicine Research Center, Royan Institute for Reproductive Biomedicine, ACECR, Tehran, Iran; 5 Department of Midwifery and Reproductive Health, Faculty of Medical Sciences, Tarbiat Modares University, Tehran, Iran; 6 Department of Biostatistics and Epidemiology, School of Medicine, Babol University of Medical Sciences, Babol, Iran

**Keywords:** Outcome, Repeated Implantation Failure, Sildenafil Citrate, Thin Endometrium

## Abstract

**Background:**

The aim of this study was to investigate the effects of vaginal sildenafil on the outcome of patients with at least two unsuccessful *in vitro* fertilization/intracytoplasmic sperm injection (IVF/ICSI) attempts.

**Materials and Methods:**

In this randomized placebo-controlled trial study, a total of 66 infertile women aged ≤38 years, with a history of normal ovarian reserve, two prior consecutive failed IVF/ICSI attempts, human chorionic gonadotropin (hCG) day endometrial thickness <7 mm in all prior IVF/ICSI cycles, normal endometrial appear- ance by either hysteroscopy, hysterosonography, or hysterosalpingography enrolled in this study. The conventional gonadotropin-releasing hormone (GnRH) protocol was used for ovarian stimulation. The patients were randomly divided into three groups: vaginal sildenafil (suppository-100 mg/daily), vaginal placebo/sildenafil (suppository-100 mg/daily), and vaginal placebo (suppository). Each patient underwent colour Doppler ultrasound on day 14 of their previous cycle to investigate any abnormalities in the uterus and adnexa. Endometrial thickness, echo pattern, uterine artery resistance, and pulsatility indices were recorded pre- and post-treatment. The primary outcome measures were implantation, chemical and clinical pregnancy rates. For data analysis, SPSS version 20 software was used. In all tests, the significance level was considered less than 0.05.

**Results:**

There was no significant difference between three groups in endometrial thickness on the hCG injection day. The chemical pregnancy in women who received sildenafil (alone or in combination with placebo) showed a two-fold increase in comparison to the placebo group. This increase was clinically meaningful, but according to sample size, it was statistically non-significant. The results of our study showed that the implantation was higher in women who received placebo/sildenafil compared to the other groups. The abortion rate was not statistically significant among the groups.

**Conclusion:**

Vaginal sildenafil may conceivably improve chemical pregnancy rates in repeated IVF failure patients. Further randomized clinical trials using oral or vaginal sildenafil with higher sample size are recommended (Registra- tion number: NCT03192709).

## Introduction

Successful implantation of an embryo requires a receptive endometrium, a good quality embryo, and embryoendometrial synchronization in all species (1). 

Endometrial receptivity during the implantation window is often assessed by ultrasonography markers such as endometrial thickness, echogenic pattern, blood flow, and biochemical markers (2). An appropriately thickened endometrium is a crucial factor for embryo implantation and can predict pregnancy outcome with high sensitivity and specificity (3). Moreover, several studies have reported a significant positive correlation between a triple-layered thickened endometrium of 7 mm (preferably >9 mm) and pregnancy rate (3-5). 

Despite significant advances in ovarian stimulation protocols, treatment of repeated, unresponsive thin endometrium is still a challenge in assisted reproductive cycles, which usually results in cycle cancellation or repeated implantation failures (6). 

There is a growing body of evidence that the endometrial growth has a relationship with the state of uterine blood flow (2). Treatment options, such as low-dose aspirin, oestradiol administration and gonadotropin therapy, low dose human chorionic gonadotropin (hCG), vitamin E, pentoxifylline, L-arginine, luteal phase support with gonadotropin-releasing hormone (GnRH) agonist, intrauterine granulocyte colony-stimulating factor (G-CSF), vaginal sildenafil, and recent application of stem cell therapy are suggested for management of a thin lining endometrium (7). 

Sildenafil citrate (Viagra®, Pfizer, NY, USA) is a 5-phosphodiestrase inhibitor that increases smooth muscle relaxation and vasodilation by preventing cGMP breakdown (8). Sildenafil citrate potentiates uterine blood flow and, in conjunction with oestrogen, it leads to the oestrogeninduced proliferation of the endometrium (9). 

It has been reported that vaginal sildenafil significantly reduced peripheral natural killer cell (NK-cell) activity and improved successful pregnancy rates in women with histories of recurrent miscarriages. Although the mechanism of influence by sildenafil on natural killer cell activity is unclear, it seems that enhancement of uterine artery flow has an effective influence on the local endometrial NK-cell population (10). However, adverse effects that include myocardial infarctions and strokes have been associated with this drug in the normal healthy population (11, 12).

This comparative pilot study evaluated the effect of vaginal sildenafil suppositories on endometrial proliferation and IVF outcome in infertile patients with a history of repeated IVF failure.

## Materials and Methods

### Patients

This phase II randomized, double-blind, placebo-controlled trial was performed at Royan Institute, Reproductive Biomedicine Research Center, Tehran, Iran, between February 14, 2014 and November 14, 2016 (NCT03192709). The assessors and patients were not aware of the treatment allocated. The Institutional Review Board and Ethics Committee of Royan Institute, Tehran, Iran reviewed and approved this study in compliance with the Declaration of Helsinki (EC/88/1045). Informed consent was obtained from all patients prior to their participation in the study.

The study population consisted of 66 infertile women aged ≤38 years. The inclusion criteria was met when the women had normal ovarian reserve [blood anti-mullerian hormone (AMH) levels >1.5 ng/mL] with at least two prior cycles with follicle stimulating hormone (FSH) <10 mIU/ml; a history of two prior consecutive failed IVF/ ICSI attempts with at least a transfer of two good quality fresh or frozen-thawed embryos; hCG day endometrial thickness <7 mm in all prior IVF/ICSI attempts; and normal endometrial appearance according to either hysterosonography, hysterosalpingography, or hysteroscopy. Women were excluded if they had a history of myomectomy or Asherman’s syndrome.

### Treatment cycle

A preliminary colour Doppler transvaginal sonography with a 4-8 MHz probe (ProSound Alpha 10; Aloka, Japan) was performed by an expert radiologist on day 14 of the patient’s prior menstrual cycle to investigate the uterine and adenexes for any abnormal findings. The endometrial parameters of endometrial thickness, endometrial pattern, pulsatility index (PI), and resistance index (RI) were measured. The uterine artery PI and RI were obtained through flow velocity waveforms from the ascending branch of the uterine artery at the point near to the internal cervical orifice and calculated as previously described (13). In the subsequent cycle, ovarian stimulation was performed with the long protocol using a GnRH agonist (14).

In the IVF treatment cycle, the patients were randomly assigned to three groups according to a random allocation sequence generated by a randomized block design. The size of each block was 3. In group A, the sildenafil (vaginal suppositories, 100 mg/day, Parnian Daroo, Co., Tehran, Iran) were administered from the first day of the FSH injection until the day of oocyte retrieval. In group B, placebo (vaginal suppositories, Parnian Daroo, Co., Tehran, Iran) was initiated from the first day of the HMG injection until 2 days before the hCG injection, after which sildenafil vaginal suppositories were initiated and continued until the day of oocyte retrieval. In group C, the placebo was given from the first day of HMG injection until the day of oocyte retrieval. Participants received 10 000 IU of hCG (Choriomon, IBSA, Switzerland) when at least two dominant follicles were 18 mm in diameter. Endometrial thickness, pattern, PI and RI were measured on the day of hCG administration and compared with the data obtained in the previous cycle without sildenafil citrate treatment. Oocytes were retrieved 36 hours later via transvaginal ultrasound-guided needle aspiration.

Embryo transfer was performed after 48 hours of oocyte retrieval. Progesterone in oil (100 mg, IM daily) or intravaginal progesterone (400 mg, twice daily) was used for luteal support and maintained until the pregnancy test was conducted. Serum hCG levels were measured on the 14^th^ day following oocyte retrieval. Vaginal ultrasound confirmation of pregnancy was performed at 4-6 weeks after embryo transfer.

A chemical pregnancy was determined by a positive ß-hCG test result. A clinical pregnancy was confirmed by ultrasonography visualization of one or more gestational sacs or definitive clinical signs of pregnancy. The spontaneous abortion was defined as a pregnancy loss of an intrauterine pregnancy before 22 weeks’ gestation (15). 

Suppositories containing 100 mg of sildenafil were prepared from the oral tablets by a local pharmacy (Parnian Daroo, Iran). We defined the endometrial thickness threshold cut-off of <7 mm as a thin endometrium based on other studies (8).

### Statistical analysis

Data are expressed as mean ± standard error (SE) and proportion. Continuous and categorical outcome variables were compared between three intervention groups by one-way analysis of variance (ANOVA) and the chi-square test. All statistical analyses were performed using SPSS version 22 for Windows 7 (IBM Analytics, Armonk, NY). The significance level was set at 0.05. 

## Results

**Fig 1:**
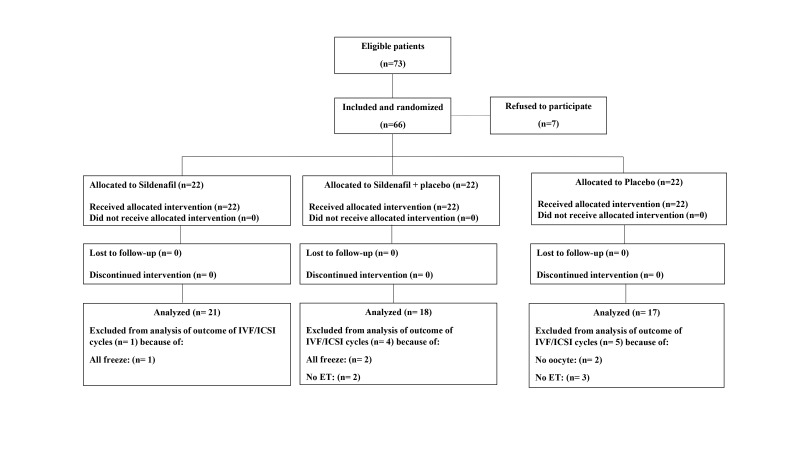
Flowchart of participants in this randomized controlled trials. IVF/ICSI; *In vitro* fertilization/intracytoplasmic sperm injection and ET; Embryo transfer

During the recruitment process, we enrolled 66 patients and allocated 22 patients to each study group. A total of 10 patients were lost to follow up for measuring clinical pregnancy for the following reasons: all of the embryos of their treatment cycle were cryopreserved (n=3), they had no oocytes at retrieval (n=2), or no embryos to transfer (n=5). The flow diagram explicitly shows the number of participants at the beginning, intervention allocation, follow-up, and analysis (Fig .1).

Table 1 shows the baseline characteristics of the three study groups. There were no significant differences among the study groups. The majority of patients had normal endometrial patterns, which were similar between the three study groups (Table 1). Uterine artery PI and RI in the left and right were not statistically significant among the study groups (P>0.05). Table 1 shows the results of the ovulation stimulation cycle and include initial endometrial thickness, number and type of ampoules used, time interval for ovulation stimulation, and numbers of total and metaphase II (MII) oocytes. These parameters did not differ significantly among the three intervention groups. Table 2 shows numbers of embryos transferred in total and by grade (A, B and C). These values were not different among the groups. The generated embryos were graded as good (A and B) or poor (C) according to their morphological features, cleavage stage, multi-nucleation, equal size blastomeres, and fragmentation rate (16). 

Of note, the embryo transfer days were similar between the three groups. All of the embryos were transferred either two or three days after ovum pickup.

### Comparisons after the interventions

The endometrial thickness and patterns after the interventions were not statistically different between the study groups. Additionally, the three intervention groups were not different in left and right uterine artery PI and RI. Implantation rate was not statistically significant over the three groups (P=0.290). Clinical pregnancy rates were 33.3 (sildenafil), 33.3 (sildenafil+placebo), and 17.6 (placebo) as seen in Table 2. Although the clinical pregnancy rates varied among the intervention groups, they were not statistically different (Table 3). Vaginal administration of sildenafil had no adverse effects on the patients during the study.

**Table 1:** Comparison of baseline characteristics and cycle related factors between the sildenafil, sildenafil + placebo, and placebo groups prior to intervention


Variable		Sildenafil n=22	Sildenafil+placebo n=22	Placebo n=22	P value

Age (Y)		33.2 ± 4.6	31.7 ± 4.8	32.8 ± 4.6	0.568
BMI (kg/m^2^)		24.7 ± 3.7	26.2 ± 3.6	25.2 ± 2.9	0.379
Infertility duration		8.8 ± 4.9	10.5 ± 5.1	8 ± 4.1	0.220
Infertility type					
	Primary	19 (86.4)	20 (90.9)	17 (77.3)	
	Secondary	3 (13.6)	2 (9.1)	5 (22.7)	0.438
Infertility reason					
	Tubal factor	1 (4.5)	1 (4.5)	1 (4.5)	
	Male factor	14 (63.6)	11 (50)	11 (50)	
	Endometriosis	0 (0)	0 (0)	1 (4.5)	0.910
	Unexplained	1 (4.5)	2 (9.1)	1 (4.5)	
	Two or more	6 (27.3)	8 (36.4)	8 (36.4)	
Endometrial pattern					
	Normal	18 (81.8)	18 (81.8)	19 (86.4)	
	Heterogenic	2 (9.1)	3 (13.6)	3 (13.6)	0.683
	Ecogene	2 (9.1)	1 (4.5)	0	
Uterine artery PI					
	Right	2.4 ± 0.7	2.5 ± 0.7	2.9 ± 0.9	0.134
	Left	2.5 ± 0.8	2.7± 0.9	2.9 ± 1	0.440
Uterine artery RI					
	Right	80.1 ± 19.3	69.7 ± 32.7	74.2 ± 31.2	0.474
	Left	76.5 ± 25.2	66.4 ± 35.7	74 ± 31	0.527
Endometrial thickness		8.0 ± 2.4	8.9 ± 2.0	7.6 ± 2.1	0.146
Type of gonadotropins					
	FSH (75 IU/mL)	5 (22.7)	8 (36.4)	3 (13.6)	0.209
	FSH+LH (75 IU/mL)	17 (77.3)	14 (63.6)	19 (86.4)	
	Ovulation duration	9.9 ± 2.1	10.3 ± 2.2	9.1 ± 1.3	0.100
	Ampoules (n)	9.1 ± 12.2	9.6 ± 14.6	7.8 ± 8.5	0.871
	Oocytes (n)	11.5 ± 5.6	11.6 ± 6.7	8.1 ± 5.5	0.098
	MII (n)	9.3 ± 5.1	9.4 ± 5.8	6.3 ± 4.1	0.079


BMI; Body mass index, PI; Pulsatility index, FSH; Follicle stimulating hormone, LH; Luteinizing hormone, RI; Resistance index, and MII; Mature metaphase II.

**Table 2:** Comparison of treatment cycle outcomes between the sildenafil, sildenafil+placebo, and placebo groups after intervention


Variable		Sildenafil n=22	Sildenafil+placebo n=22	Placebo n=22	P value

Endometrial pattern					
	Normal	17 (77.3)	21 (95.5)	20 (90.9)	
	Heterogenic	3 (13.6)	0	2 (9.1)	0.263
	Ecogene	2 (9.1)	1 (4.5)	0	
Uterine artery PI					
	Right	2.1 ± 0.6	2.2 ± 0.7	2.0 ± 0.5	0.515
	Left	2.2 ± 0.4	2.3 ± 0.7	2.1 ± 0.3	0.357
Uterine artery RI					
	Right	79.8 ± 7.0	82.3 ± 5.9	80.0 ± 5.0	0.318
	Left	78.2 ± 18.1	79.0 ± 18.2	81.5 ± 4.0	0.747
				
Endometrial thickness		10.1 ± 2.4	10.30 ± 2.5	9.60 ± 2.5	0.656
Embryo (n)		4.8 ± 3.3	4.80 ± 2.7	3.60 ± 3.2	0.322
ET (n)		2.5 ± 1.2	2.20 ± 1.4	2.10 ± 1.6	0.603
ET grade					
	A	2.1 ± 2.0	2.13 ± 2.3	2.14 ± 2.2	>0.999
	B	1.0 ± 1.0	1.0 ± 1.1	1.0 ± 1.1	0.957
	C	0.4 ± 0.8	0.10 ± 0.3	0.40 ± 1.0	0.555


PI; Pulsatility index, RI; Resistance index, and ET; Embryo transfer.

**Table 3:** Comparison of reproductive outcomes between the sildenafil, sildenafil+placebo, and placebo groups after intervention


Variable	Sildenafil n=21	Sildenafil+placebo n=18		Placebo n=17	P value

Chemical pregnancy	7/21 (33.3)		6/18 (33.3)	3/17 (17.6)	0.490
Clinical pregnancy	4/21 (19.0)		6/18 (33.3)	3/17 (17.6)	0.464
Implantation rate	5/56 (8.9)		8/51 (15.7)	3/47 (6.4)	0.290
Miscarriage rate	0		1 (16.7)	0	0.562


Data are presented as n (%).

## Discussion

The importance of the endometrial pattern as a predictor of treatment cycle outcome in IVF-treated patients is well-documented (17). In addition, endometrial quality is also an important factor for the successful implantation of the foetus (18). Endometrial thickness increases with high pregnancy rates; however, pregnancy rates are not predictable only based on endometrial thickness (19). Uterine arterial blood flow seems to affect endometrial growth and the outcome of pregnancy. Studies have shown that sildenafil widens the vasculature by its effects on the smooth muscles of the arteries (10, 20). Sildenafil citrate is an inhibitor-5-phosphodiestrase type that, by preventing the effect of cGMP, exacerbates the effect of NO on the smooth muscle of the arteries (20, 21). According to the results of some studies, the use of sildenafil during the proliferative phase of the cycle improves uterine blood flow and endometrial growth, and results in a higher level of implantation and pregnancy in patients with repeated IVF failure and Ashermen syndrome (8, 22). Also the administration of sildenafil (50 mg intravenous) in a sterilized animal model (sheep) has been shown to exacerbate uterine flow (23). Several studies have shown a correlation between ‘‘thin endometrium’’ and low implantation rates (9, 24). The results of current study showed increased endometrial thickness in the three groups on the day of hCG injection, but the increase was not statistically significant. The results of previous studies have shown that sildenafil citrate (vaginal or oral alone or with oestradiol) is significantly effective in improving endometrial thickness (25-27). Sher and Fisch (22) reported that the use of sildenafil vaginal suppository could reduce the adverse effects of headache and low blood pressure compared with oral sildenafil. In another study, they reported that vaginal sildenafil (25 mg, 4 times per day) improved endometrial thickness (≥9 mm) in 70% of the patients (8). In a prospective study, Takasaki et al. (25) compared the effects of vaginal vitamin E, L-arginine, and vaginal sildenafil citrate on endometrial thickness in patients with endometrial thickness less than 8 mm and right arterial resistance in their radial vessels (RA-RI ≥0.81). The results showed that vitamin E, L-arginine, and sildenafil citrate significantly improved RA-RI and endometrial thickness in these patients; however, the improvement in endometrial thickness in patients treated with sildenafil citrate was more than the other two groups. Several studies (26-28) showed that vaginal sildenafil significantly improved endometrial thickness in patients with history of poor endometrial thickness in the previous cycles. Jerzak et al. (10) reported that endometrial thickness significantly increased after administration of oral sildenafil (25 mg, 4 times per day) in women with a history of abortion. Dehghani Firouzabadi et al. (29) recommended oral sildenafil administration as an appropriate solution for improving endometrial admission in patients with unsuccessful cycles from low endometrial thickness. Their results showed that the triple line endometrial pattern in the sildenafil citrate + oestradiol group was significantly higher than in the oestradiol-only group, while the intermediate pattern of the endometrium was not significantly different between the two groups. Fetih et al. (30) reported that sildenafil vaginal gel significantly increased endometrial thickness and uterine blood flow, and might improve pregnancy rate in patients with clomiphene citrate (CC) failure due to thin endometrium. The results reported by Chanona et al. (31) showed that the use of vaginal sildenafil in patients whose endometrial thickness was equal to or less than 7 mm in the failed assisted reproductive technology (ART) cycles led to an increase in implantation and pregnancy. Zinger et al. (32) reported that two patients with a history of curettage and secondary infertility were treated with sildenafil after removing adhesions by surgery (due to thin endometrial thickness in previous IVF cycles), and both patients became pregnant during the first cycle of sildenafil administration. Increases in their endometrial thicknesses was also shown in transvaginal ultrasonography.

Several treatment modalities have been offered to patients with ‘‘thin’’ endometrium, including hormonal manipulation by oestrogen and gonadotropin therapy, low-dose hCG, tamoxifen, L-arginine or sildenafil, vitamin E, pentoxifylline, low-dose aspirin, hysteroscopic adhesiolysis, intrauterine infusion of growth factor such as G-CSF, and the recent application of regenerative medicine. Despite the large variety of treatment, most options lead to only minor modifications in the endometrium thickness and subsequent pregnancy, and when this modality fails, patients are eventually candidates for surrogacy. Treatment of thin endometrium remains a challenge and future investigations are required to further clarify and ideally manage patients with thin endometrium (7). 

In the present study, the clinical and chemical pregnancy rates in the two groups of women who were taking sildenafil alone and the women who took placebo and sildenafil showed a twofold increase compared to the placebo-only-treated women, which according to the sample size, this increase is not significant. The results reported by Dehghani Firouzabadi et al. (29) in a study of 80 patients showed that the chemical pregnancy rate was higher but not statistically significant in the patients who used sildenafil; this finding was consistent with the results of our study. However, AbdelKader Fahmy et al. (33), with a sample size of 70 patients, reported a significantly greater chemical pregnancy rate in the sildenafil group.

In this study, the clinical pregnancy rate was higher in the sildenafil and sildenafil+placebo groups than in the placebo group. Although this increase was not statistically significant, it was clinically shown to be twofold. These results were consistent with the findings of AbdelKader Fahmy et al. (33) and Kim et al. (28). The study by AbdelKader Fahmy et al. (33) reported a 2.5-fold increase in pregnancy in the sildenafil group, but this difference was not statistically significant. Kim et al. (28) reported that the use of vaginal sildenafil plus oral oestrogen pills in the luteal phase of patients treated with an IVF-ET cycle increased the pregnancy rate by two-fold, but this increase was not statistically significant. Mangal and Mehirishi (34) showed that the rate of pregnancy in the patients who used vaginal sildenafil in three successive intra uterine insemination (IUI) cycles was significantly higher in the third cycle than in the group who used oestradiol valerate, while this difference was not significant in the first and second cycles. The findings from a retrospective study by Margreiter et al. (35) showed a significant improvement in the rate of implantation, endometrial thickness and pregnancy, and decreased abortion in the group of patients treated with vaginal sildenafil.

The endometrial receptivity is an important stage in the ART cycles, the results of our study showed a higher rate of implantation in the women who took sildenafil+placebo than in the other two groups, which was consistent with the results of Dehghani Firouzabadi et al. (29).

In the present study, 2 out of 7 cases of pregnancy ended in abortion in the sildenafil group, 1 out of 6 cases of pregnancy in the sildenafil+placebo group, and 0 of 3 cases of pregnancy in the placebo group. The rate of abortion was negligible in the sildenafil group. Follow-up evaluations showed a molar pregnancy in the sildenafil group. Dzieciol et al. (36) reported that sildenafil citrate increased uterine tissue perforation during the uterus preparation for fertility. 

In our study, higher numbers of MII oocytes were retrieved in the sildenafil groups compared to the placebo group, which was clinically important; however, one-way ANOVA results showed that this finding was not statistically significant. To our knowledge, there is only one study (abstract available) by Vidal et al. (37) that provided evidence of the benefit of sildenafil supplementation in the first days of hyperstimulation induction in terms of the numbers of mature and fertilized oocytes. Therefore, more research on this topic needs to be undertaken. 

## Conclusion

Vaginal sildenafil might conceivably improve chemical and clinical pregnancy rates in repeated IVF failure patients. Since this was a pilot study, we recommend that clinical trials should be conducted with vaginal or oral sildenafil on larger numbers of these patients.
